# Liprins in oncogenic signaling and cancer cell adhesion

**DOI:** 10.1038/s41388-021-02048-1

**Published:** 2021-10-15

**Authors:** Henna Pehkonen, Ivan de Curtis, Outi Monni

**Affiliations:** 1 Applied Tumor Genomics Research Program and Department of Oncology, Faculty of Medicine, University of Helsinki, Helsinki, Finland; 2 Cell Adhesion Unit, Division of Neuroscience, IRCSS San Raffaele Scientific Institute, San Raffaele Vita-Salute University, Milan, Italy; 3 iCAN Digital Precision Cancer Medicine Flagship, Helsinki, Finland

**Keywords:** Oncogenes, Focal adhesion

## Abstract

Liprins are a multifunctional family of scaffold proteins, identified by their involvement in several important neuronal functions related to signaling and organization of synaptic structures. More recently, the knowledge on the liprin family has expanded from neuronal functions to processes relevant to cancer progression, including cell adhesion, cell motility, cancer cell invasion, and signaling. These proteins consist of regions, which by prediction are intrinsically disordered, and may be involved in the assembly of supramolecular structures relevant for their functions. This review summarizes the current understanding of the functions of liprins in different cellular processes, with special emphasis on liprins in tumor progression. The available data indicate that liprins may be potential biomarkers for cancer progression and may have therapeutic importance.

## Introduction

Liprins belong to the LAR (leukocyte common antigen related) protein tyrosine phosphatase interacting protein family [1]. In mammals, the liprin family consists of liprin-α (α1, α2, α3, α4), β (β1, β2) proteins, which are well conserved in evolution [1, 2], and kazrin E, a protein regulating desmosome assembly and tissue morphogenesis [3–5]. *C. Elegans* has one homolog of liprin-α (*syd-2*, synapse defective-2) and one homolog for liprin-β (*hlb-1*, liprin-β homolog) [1, 6]. In *Drosophila* there are single liprin-α (Dliprin-α), liprin-β and liprin-γ homologs; both liprin-β and liprin-γ interact with liprin-α [1, 7, 8]. In *Drosophila*, liprin-γ is thought to counteract the activity of liprin-α and liprin-β proteins in synapse formation [7]. The carboxyterminal part of liprin-α and liprin-β proteins include a highly conserved region containing three SAM (sterile-alpha motif) domains, which are important in protein–protein interactions [1, 2, 4]. Liprin-α proteins interact with the intracellular part of the transmembrane LAR and receptor tyrosine phosphatases included in this subfamily (LAR-RPTPs) with C-terminal SAM domains [1, 2, 4, 9], which are also responsible for liprin α/β binding [1, 4]. Despite forming hetero-oligomers with liprin-α, liprin-β has not been shown to bind LAR [1]. The N-terminal region of all liprins is characterized by coiled coil structures, which are responsible for homodimerization of liprin-α and liprin-β proteins, as well as for heterodimerization of liprin-α proteins with other liprin-α subfamily members [1, 2, 4].

Liprin-α1 is the only liprin-α having widely distributed mRNA and protein expression within different tissues [1, 10–12], whereas liprin-β’s are more broadly expressed [1] (Table 1). Liprin-α2 and -α3 are predominantly expressed in the brain tissue, whereas liprin-α4 is expressed in the heart and muscle tissues as well [1, 10, 12]. Although all four liprin-α proteins are expressed in the brain tissue, they show somewhat different brain tissue distribution and expression level [11]. Liprin-α2 and -α3 show strong expression throughout the brain, while liprin-α1 is expressed at lower levels with the exception of the olfactory bulb and the cerebellum. Liprin-α4 shows strongest expression in the cerebellum [11, 12]. Both the liprin-α1 and liprin-α3 show expression in astrocytes [11, 12], suggesting liprins may have a role in glial cells based on their expression.

**Table 1:** Expression of liprins both on the mRNA and protein level.

Gene human	Gene mouse	Protein	Organism	mRNA/protein	Tissue	Specific brain tissue	Method	Author, Ref.
*PPFIA1*	*ppfia1*	Liprin-α1	Human	mRNA	Heart, brain, placenta, lung, liver, skeletal muscle, kidney, pancreas		Northern blot	Serra-Pagès et al. [1]
			Rat	mRNA	Brain, lung, heart, muscle, liver, spleen, kidney, thymus, testes	OB, CB, (other parts weak)	qPCR	Zürner and Scoch [10]
			Rat	Protein	Brain	CTX, OB, HC, CB, glial cells	Western blot, IHC, IF	Spangler et al. [11]
			Mouse	Protein	Brain, lung, heart, muscle, liver, kidney, spleen, testes	OB, CB, (other parts weak), glial cells	Western blot, IHC, ICC	Zürner et al. [12]
			Rat	mRNA/protein	Brain	HC, CB	ISH, ICC	Zürner et al. [12]
			Embryonic mouse	Protein	Brain	Developmental stages	Western blot	Zürner et al. [12]
*PPFIA2*	*ppfia2*	Liprin-α2	Human	mRNA	Brain		Northern blot	Serra-Pagès et al. [1]
			Rat	mRNA	Brain, liver, testes	OB, STR, CTX, HC, TH, MB, CB, BS	qPCR	Zürner and Scoch [10]
			Rat	Protein	Brain	CTX, OB, HC, CB	Western blot, IHC, IF	Spangler et al. [11]
			Mouse	Protein	Brain, testes	OB, STR, CTX, HC, TH, MB, CB, BS	Western blot, IHC, ICC	Zürner et al. [12]
			Rat	mRNA/protein	Brain	OF, CTX, HC, MB	ISH, ICC	Zürner et al. [12]
			Embryonic mouse	Protein	Brain	Developmental stages	Western blot	Zürner et al. [12]
*PPFIA3*	*ppfia3*	Liprin-α3	Human	mRNA	Brain		Northern blot	Serra-Pagès et al. [1]
			Rat	mRNA	Brain, thymus, testes	OB, STR, CTX, HC, TH, MB, CB, BS	qPCR	Zürner and Scoch [10]
			Rat	Protein	Brain	MB, CTX, OB, HC, CB, glial cells	Western blot, IHC, IF	Spangler et al. [11]
			Mouse	Protein	Brain	OB, STR, CTX, HC, TH, MB, CB, BS	Western blot, IHC, ICC	Zürner et al. [12]
			Rat	mRNA/protein	Brain	OF, CTX, HC, MB	ISH, ICC	Zürner et al. [12]
			Embryonic mouse	Protein	Brain	Developmental stages	Western blot	Zürner et al. [12]
*PPFIA4*	*ppfia4*	Liprin-α4	Human	mRNA	Heart, brain, skeletal muscle		Northern blot	Serra-Pagès et al. [1]
			Rat	mRNA	Brain, muscle, testes	CB	qPCR	Zürner and Scoch [10]
			Rat	Protein	Brain	MB, CTX, OB, HC, CB, BS, spinal cord	Western blot, IHC, IF	Spangler et al. [11]
			Mouse	Protein	Brain	OB, STR, CTX, HC, TH, MB, CB, BS	Western blot, IHC, ICC	Zürner et al. [12]
			Rat	mRNA/protein	Brain	HC, CB	ISH, ICC	Zürner et al. [12]
			Embryonic mouse	Protein	Brain	Developmental stages	Western blot	Zürner et al. [12]
*PPFIBP1*	*ppfibp1*	Liprin-β1	Human	mRNA	Heart, brain, placenta, lung, skeletal muscle, kidney, pancreas		Northern blot	Serra-Pagès et al. [1]
*PPFIBP2*	*ppfibp2*	Liprin-β2	Human	mRNA	Heart, brain, placenta, lung, liver, skeletal muscle, kidney, pancreas		Northern blot	Serra-Pagès et al. [1]

*OB* olfactory bulb, *STR* striatum, *CTX* cortex, *HC* hippocampus, *TH* thalamus, *MB* midbrain, *CB* cerebellum, *BS* brain stem, *IHC* immunohistochemistry, *IF* immunofluorescence, *ICC* immunocytochemistry, *ISH* in situ hybridization.

Liprin-α proteins show spatiotemporal expression patterns during mouse brain development [12]. Liprin-α1 is the only member with different temporal and spatial expression pattern as compared to the other liprin-α protein members [12]. Ppfia1 is homozygously lethal, and heterozygous animals display significant changes in phenotypes including abnormalities in the vocalization and abnormal coat/hair pigmentation. On the other hand, ppfia2, ppfia3, ppfia4 and ppfibp2 KO mice are homozygously viable with ppfia2, ppfia3, and ppfibp2 KO mice showing various changes in phenotypes such as abnormal retinal vasculature morphology, vocalization, increased activity, and heart rate (www.mousephenotype.org) [13, 14]. Of note, liprins contain protein regions that are predicted to be intrinsically disordered [15, 16]. Intrinsically disordered regions are included in several proteins that are involved in the formation of multimolecular assemblies defined as biomolecular condensates, which are formed by liquid–liquid phase separation (LLPS) [17–19]. In addition, LLPS and biomolecular condensates may be important in cancer and in small molecule therapeutics [20, 21]. In this respect, very recent findings have shown that liprins may contribute to the assembly of biomolecular condensates [22–24], suggesting that both specific protein–protein interactions as well as LLPS–mediated condensates may contribute to essential physiological processes as well as play a role in different signaling pathways critical to tumor cell progression. In this review, we are focusing on recent findings on the involvement of liprins in oncogenic signaling after presenting an overview of the crucial role of liprins in neuronal cells.

### The function of liprins in the nervous system and interaction with kinesins

**Fig. 1:**
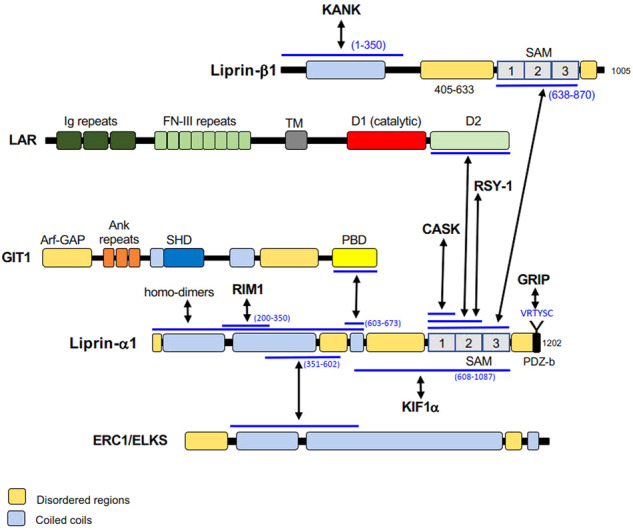
Liprin-α proteins interact with several partners by specific protein–protein interactions. Liprin-α1 is shown in the drawing. Black arrows show direct interactions between the indicated proteins/protein regions (blue lines; residues in brackets). Ank ankyrin, Arf-GAP GTPase activating protein for Arf GTPases, D1 phosphatase domain, D2 phosphatase–like domain (catalytically inactive), FN-III fibronectin type III, Ig immunoglobulin, PBD paxillin binding domain, PDZ-b, PDZ binding peptide, SAM sterile-alpha motif, SHD Spa2 homology domain, TM transmembrane region. Intrinsically disordered regions were identified by the program DisEMBL [18], coiled coil regions by the program COILS [19].

Liprin-α proteins bind to several partners with their coiled coil and SAM domains (Fig. 1). At the presynaptic sites, liprin-α proteins display widespread functions in synapse assembly as well as in presynaptic organization. In addition to presynaptic sites, liprin-α proteins are localized in dendrites, and contribute to the maturation of the postsynaptic sites [6, 25–31]. Liprin-α proteins bind to MALS and CASK proteins, which are multidomain scaffolding proteins highly expressed in the mammalian nervous system [32–35]. Liprin-α proteins interact also with RIM1 [36], ERC1/ELKS [37, 38], KIF1A [26], GRIP and GIT1 [25, 27, 39] to organize presynaptic active zones and regulate neurotransmitter release. In synapses, liprin-α1 is regulated by either CAMKII or ubiquitin-proteasome mediated degradation, and liprin-α1 degradation by CAMKII is important for correct LAR distribution and dendritic development [40]. Liprin-α2 is important in promotion of protein dynamics in active zone of hippocampal synapses, and ultrastructural analysis has shown that this protein regulates the presynaptic organization and the size of the synaptic vesicle pool [35]. In mammals, liprin-α3 knockout mice show secretory impairment and defects in presynaptic active zone due to the prevalent role of liprins in synapse assembly. While liprin-α2 and -α3 may compete for positioning at the active zone, liprin-α2 cannot fully compensate the presynaptic loss of liprin-α3 [14]. Knockout of both the liprin-α2 and -α3 lead to decreased number of synaptic vesicles, reduced levels of presynaptic proteins, as well as reduction of docked vesicles in the active zone of primary mouse hippocampal neurons, which further points to the conclusion, that liprin-α2 and -α3 are necessary for presynaptic composition and normal active zone structure [23].

In *C. elegans* the liprin-α homolog SYD-2 is an important player in the organization of the presynaptic active zone [6]. SYD-2 interacts with RSY-1, which negatively regulates SYD-2 and the formation of synapses [41]. SYD-2 recruits multiple synaptic components to presynaptic sites and plays a major role in the architecture of the neuromuscular junctions as well as in the assembly and stabilization of presynaptic sites [41, 42]. SYD-2 mutants display morphological changes at the presynaptic active zone and defects in presynaptic assembly that are due to problems in the oligomerization of liprin-α proteins [6, 42, 43]. Similarly, the *Drosophila* Dliprin-α is needed for synaptic morphogenesis and synaptic vesicular transport [8, 44]. Dliprin-α mutants display defects in synaptic morphology and photoreceptor target selection at presynaptic sites [8, 44, 45]. In *C. elegans* and *Drosophila*, the interaction between SYD-2/Liprin-alpha and SYD-1/syd-1 (RhoGAP-100F), a Rho GTPase activating protein, is important for proper localization of SYD-2/liprin-α at presynaptic sites allowing SYD-2/liprin-α to execute its active zone assembly function [30, 46, 47], as well as for proper recruitment of the synaptic proteins [43, 47, 48]. SYD-1/syd-1 acts as a positive regulator upstream of SYD-2/liprin-α. Previous data suggest, that SYD-1 and SYD-2 (liprin-alpha) are not absolutely dependent on each other to localize to presynaptic sites, but SYD-2 overexpression can overcome the defects of mutant SYD-1 [43, 47].

Several studies suggest interactions of liprins with kinesin motor proteins [26, 49, 50]. Liprin-α1 interacts with kinesin KIF1A and functions as a KIF1A receptor during transport of membrane, signaling, and cytoskeletal proteins via microtubule tracks [26]. Liprin-α1 is also important for the hedgehog signaling dependent trafficking of Kif7 and Gli proteins to the tips of cilia during the embryonic development. Liprin-α1 interacts with PP2A and promotes the dephosphorylation of Kif7. Loss of liprin-α1 leads to defective development in zebrafish embryo [49]. KIF21A suppresses microtubule growth at the cell cortex and contributes to microtubule organization at the cell edge. KIF21A is recruited to the cell cortex by KANK, which interacts with liprin-α1/β1 complex. Liprin-α1, liprin-β1, KANK1, and KIF21A co-operate with LL5β, ELKS, and CLASPs in cortical microtubule organization. LL5β and KANK1 are required for targeting of the microtubule binding proteins such as CLASPs and KIF21A, whereas liprins and ELKS play a role in scaffolds required for protein clustering. While all these proteins co-operate in microtubule organization, they have different turnover rates and dynamics at the cell cortex, which is a similar situation than that found in focal adhesions [50].

In summary, liprins act as scaffolds for assembling very large protein complexes involved in the regulation of synaptic signaling and assembly [7, 14, 29]. In addition to the established role of liprins in regulating these essential neuronal functions, liprins have recently received more attention for their role as regulators of the motility and invasion of cancer cells [4, 5, 51–53] (Table 2), as discussed in the next part of this review.

**Table 2:** Function of liprins in experimental model systems.

Organism/cell type	Protein	System/cells	Manipulation	Liprin function	Author	Ref.
Organism						
C. Elegans	SYD-2/liprin-α	Presynaptic active zone	syd-2 mutants, loss/gain-of-function	Presynaptic vesicles, synaptic assembly	Kittelman et al.	[42]
	SYD-2/liprin-α	Presynaptic termini	syd-2 mutant, loss-of-function	Differentiation of presynaptic termini, synaptic transmission	Zhen and Jin	[6]
	SYD-2/liprin-α	Presynaptic active zone	syd-2 mutant, gain-of-function	Organization of presynaptic active zone	Dai et al.	[37]
	SYD-2/liprin-α	Presynaptic active zone	syd-2 mutant	Presynaptic assembly	Taru and Jin	[43]
Drosophila	Dliprin-α	Synapse/axon	Liprin-α mutant, loss-of-function	Promoting delivery of synaptic components	Miller et al.	[44]
	Dliprin-α	Synapse	Liprin-α mutant, loss-of-function	Normal synaptic morphology	Kaufmann et al.	[8]
	Dliprin-α	Axon	Liprin-α mutant	Photoreceptor target selection, axon extension toward target	Choe et al.	[45]
Mice	Liprin-α3	Hippocampal synapses	Knockout mice	Synapse assembly	Wong et al.	[14]
Mice	Liprin-α2, liprin-α3	Hippocampal neurons	Knockout mice/double knockout	Active zone composition and ultrastructure, neurotransmitter release	Emperador-Melero et al.	[23]
Rat	Liprin-α2	Hippocampal neurons/synapses	shRNA	Presynaptic protein composition, regulation of synaptic vesicle pool size	Spangler et al.	[35]
Zebrafish	Liprin-α1	Vascular system	Morpholino-mediated knockdown	Vascular morphogenesis, fibrillogenesis	Mana et al.	[102]
Xenopus Laevis	Liprin-β1	Vascular system	Morpholino-mediated knockdown	Lymphatic vascular development	Norrmèn et al.	[103]
Mice	Liprin-α1	Breast cancer	siRNA	Promotion of lung metastases	Chiaretti et al.	[72]
Mice	Liprin-α1	Chronic myelogenous leukemia	siRNA	Growth of leukemic cells	Gu et al.	[135]
Type of cell line						
Breast cancer	Liprin-α1	MDA-MB-231	siRNA/OE	Promotive effect on lamellipodia stabilization, invasion/migration	Astro et al.	[53], [92]
Breast cancer, MEFs	Liprin-α1	MDA-MB-231, NIH-3T3	siRNA/OE	Promote invadosome maturation, ECM degradation	Sala et al.	[118]
Breast cancer	Liprin-α1	MDA-MB-231	siRNA/OE	Promotive effect on focal adhesion turnover and motility/invasion	Astro et al.	[90]
Breast cancer	Liprin-α1	MDA-MB-231, Hs578T	shRNA	Promotive effect on invasive growth	Pehkonen et al.	[51]
Breast cancer	Liprin-α1	Hs578T	NA	Snail binding to *PPFIA1*	Maturi et al.	[121]
cervical cancer	Liprin-α1, liprin-α3	HeLa	siRNA/OE	inhibitory effect on the formation of stress fibers	Sakamoto et al., Brenig et al.	[100], [101]
Breast cancer	Liprin-α4	MCF7	siRNA	Promotive effect on formation of cell–cell junctions	Mattauch et al.	[131]
Breast cancer	Liprin-β1	MDA-MB-231	siRNA	Promotive effect on tumor cell motility and lamellipodia stabilization	Chiaretti et al.	[72]
Breast cancer	Liprin-β2	MDA-MB-231	siRNA/OE	Inhibitory effect on migration/invasion/motility	von Thun et al.	[75]
Breast cancer	Liprin-β2	MDA-MB-231	siRNA	Inhibitory effect on migration/invasion and ECM degradation	Chiaretti et al.	[72]
HNSCC	Liprin-α1	SCC-25, UT-SCC cell lines	shRNA	Promotive effect on cohesive growth, localization to invadosomes	Pehkonen et al.	[51]
HNSCC	Liprin-α1	UT-SCC cell lines	shRNA	Promotive effect on invasive phenotype	Pehkonen et al.	[52]
HNSCC	Liprin-α1	FaDu, SCC cell lines	siRNA	Inhibitory effect on invasion/migration	Tan et al.	[120]
Colon carcinoma	Liprin-α1	RKO	siRNA/OE	Promotive effect on motility/migration/spreading	Shen et al.	[127]
Ovarian cancer	Liprin-β2	A2780-Rab25	siRNA/OE	Inhibitory effect on migration/invasion/motility	von Thun et al.	[75]
Bladder cancer	Liprin-α1	T24	siRNA	Promotive effect on progranulin-dependent motility	Buraschi et al.	[134]
Monkey kidney fibroblast like	Liprin-α1	COS-7	siRNA, OE	Promotive effect on cell spreading/migration	Asperti et al.	[91], [98]
Human embryonic kidney	Liprin-α3	HEK293T	OE	Promotive effect on formation of droplets/condensates	Emperador-Melero et al.	[23]

The table shows function of liprin both in physiological system and in cancer. In cancer cell lines, it is shown whether the impact of liprin expression has inhibitory or promotive effect.

### Genetic alterations in genes encoding liprins

**Fig. 2:**
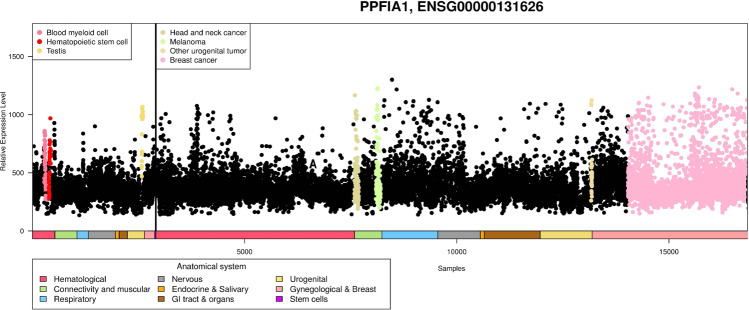
*PPFIA1* is overexpressed in head and neck and breast cancers. The figure shows a body-wide gene profile of the *PPFIA1* gene across 15392 malignant and 3082 healthy samples. Each dot represents the expression of *PPFIA1* gene in one sample. Anatomical origins of each sample are marked with colored bars below the gene plot. Sample types having higher than average expression or an outlier expression profile are additionally colored. The image has been modified from MediSapiens ISTOnline database.

Human liprin-α1 is encoded by the gene *PPFIA1* (PTPRF [Protein Tyrosine Phosphatase Receptor Type F polypeptide] Interacting Protein Alpha 1), which maps to the 11q13 chromosomal region that is often amplified in multiple cancer types, such as in head and neck squamous cell carcinoma (HNSCC), breast cancer and esophageal carcinoma [54–61]. Across different studies, the 11q13 region is amplified in 30–62% of HNSCC, in 15–20% of breast cancer and in 33% of esophageal cancers [reviewed in [57]]. The minimal common region of the amplification in HNSCC, where this amplification is the most frequent, ranges from around 0.9 to 2 Mb (amplicon core 1.5 Mb), and the amplification is associated with poor survival of the patients [57–65]. Across several cancers, *PPFIA1* is overexpressed in carcinomas including those of the head and neck and breast carcinomas (Fig. 2). In tumors, *PPFIA1* is often altered by gene amplification, whereas mutations and deletions are rare. *PPFIA1* has been recently reported to be nonsynonymously mutated in aggressive papillary thyroid microcarcinomas (PTMC) [66]. The functional consequences of these variants are currently unknown.

In addition to gene amplifications and genetic variants, few examples of gene fusions involving liprin–encoding genes have been reported. A fusion gene *PPFIBP1–ALK* between liprin-β1 and anaplastic lymphoma receptor tyrosine kinase has been reported in pulmonary inflammatory myofibroblastic tumor, and it is shown to have transforming potential in vivo in mice [67]. Fusions of *PPFIBP1* have also been reported in melanocytic tumors (*PPFIBP1-ROS1* and *PPFIBP1-MET*) [68, 69]. In addition, *PPFIBP2-BRAF* fusion has been reported in metastatic melanoma patient [70] and *PPFIBP2-RET* fusion in papillary thyroid carcinoma has shown to lead to the activation of mitogen-activated protein kinase (MAPK) pathway and growth–promoting properties [71]. In general, liprin-β2 (*PPFIBP2*) is considered to promote tumor suppressor properties opposite to liprin-β1 (*PPFIBP1*) with oncogenic functions [72]. Germline loss-of-function mutations of *PPFIBP2* have been associated with shorter survival in prostate cancer [73]. *PPFIBP2* has been proposed as one of the possible biomarkers in endometrial cancer [74] and it has been shown to display anti-invasive properties in cancer cells [72, 75]. Thus, although *PPFIA1* is so far the only gene in the liprin family which is commonly amplified in several tumor types, genetic alterations in other liprin family genes, although infrequent, have recently been found to contribute to cancer progression.

### Liprin-α interaction with LAR receptor tyrosine phosphatase

The family of receptor protein tyrosine phosphatases (RPTPs) contains multiple subfamilies [76], and the LAR-RPTP subfamily include LAR, PTPσ and PTPδ receptor tyrosine phosphatases, which interact with liprin-α proteins [1, 9, 77]. LAR protein family consists of an extracellular part with immunoglobulin-like and fibronectin III domains and a cytoplasmic part with two PTPase domains, D1 and D2 [1, 9]. The D1 domain has PTPase catalytic activity, whereas the carboxy terminal D2 domain is catalytically inactive [9, 78, 79]. Liprin-α proteins bind to LAR through the SAM domains, and they co-localize at specific sites at the proximal end of focal adhesions [1, 2]. While the interaction of liprin-α proteins to LAR occur via the cytoplasmic D2 domain [2], both D1 and D2 are required for stronger interaction [80]. Liprin-β1 does not bind to LAR, but LAR can allosterically inhibit the binding of liprin-β1 to liprin-α1 providing insights into underlying mechanisms in formation of complexes of liprin proteins [80].

The cellular localization of LAR transmembrane tyrosine phosphatase is regulated by liprin-α1 and the interaction of liprin-α1 with LAR facilitates the formation of LAR clusters [1, 2, 80]. There is evidence that association between liprin-α1 and LAR is controlled by liprin-α1 autophosphorylation [1, 81]. LAR has multiple targets and it has been shown to be involved in various signaling pathways related to phosphatase regulation [82]. Liprin-α1–promoted LAR clustering attenuates phosphatase activity, showing an interesting capacity of liprin-α1 to regulate the cellular activities of target proteins through protein–protein interactions [80]. Liprin-α1 proteins promote localization of LAR family tyrosine phosphatases at specific sites on the plasma membrane, possibly regulating their interaction with the extracellular environment and their association with substrates [1, 2, 80, 81]. When positioned in the right location at the focal adhesions with the contribution of liprin-α1 proteins, LAR has the potential to dephosphorylate proteins belonging to signaling pathways involved in the regulation of adhesion [1, 2, 4]. In cancer cells, this may lead to dephosphorylation of proteins important in oncogenic signaling. Recently, PTPRF/LAR has been shown to have both the tumor suppressive and oncogenic properties, and its expression is altered in cancer. PTPRF/LAR suppresses liver cancer tumorigenesis, and reduced expression of PTPRF/LAR leads to development of tumors [83]. However, PTPRF ectodomain was upregulated in a prostate cancer mouse model [84], as well as in metastatic breast cancer suggesting that altered expression of PTPRF/LAR is tumor type dependent [85].

### Liprins as regulators of cell adhesion

Focal adhesions are cellular multiprotein structures that link the actin cytoskeleton to the ECM via the transmembrane integrin receptors [86, 87]. The structures important in actin– and adhesion–dependent mesenchymal cell migration are the lamellipodia which arise at the leading edge of migrating cells [88, 89]. Several studies point to the importance of liprin-α1 in focal adhesions and in regulation of the turnover, size and shape of these structures [4, 53, 90–93], and liprin-α1 is part of the integrin adhesome network [94–96]. Liprin-α1 together with ERC1/ELKS and LL5 proteins (LL5α and -β) regulate focal adhesion turnover and lamellipodial dynamics near the protruding tumor cell edge [92], where these proteins are part of dynamic plasma membrane-associated platforms (PMAPs) [15]. Their dynamic behavior suggests that PMAPs are liquid–like biomolecular condensates driven by LLPS of ERC1 that can recruit liprin-α1 via specific protein–protein interactions [97].

Interestingly, recent studies have shown that among PMAP proteins, not only ERC/ELKS, but also liprin-α proteins can undergo LLPS. In *Caenorhabditis elegans*, both SYD-2/liprin-α and ELKS-1 undergo LLPS during presynaptic development [22]. SYD-2/liprin-α LLPS, which is driven by a central intrinsically disordered region of the protein, is important for synaptic function, and allows the recruitment of other players such as GIT1 via specific protein–protein interactions (Fig. 1). Also the mammalian family member liprin-α3 has recently been shown to undergo reversible LLPS following phosphorylation of serine 760 [23]. This study has shown that while the expression of the wildtype liprin-α3 can rescue the defect in synapses observed in neurons with double knockout of liprin-α2 and liprin-α3, expression of liprin-α3 with a mutation of serine 760 prevents both LLPS and the rescue of the synaptic defect in the double knockout neurons [23]. These studies suggest that altering the ability of liprin-α proteins to undergo LLPS may also influence the function of these proteins in tumor cell motility.

Liprin-α1 and ERC1 control active β1-integrin internalization through Rab7 positive endosomes [90]. Reduced liprin-α1 levels lead to an increase in the lamellipodia number and a decrease of their stability during migration, whereas overexpression of liprin-α1 increases the stabilization of the lamellipodia, and the turnover of focal adhesions at the protrusive front of breast cancer cells [53, 92]. Liprin-α1 stabilizes inactive β1 integrins at the cell membrane, and regulates β1-integrin internalization and recycling [90, 92, 93]. Liprin-α1 cooperates with the β1-integrin binding protein talin to promote cell spreading and integrin organization [91]. Moreover LAR and liprin-α1 association is needed for protrusions during cell spreading, but liprin-α1 has also independent functions related to focal adhesions [91]. Knockdown of LAR leads to decrease in cell spreading but not in invasion, whereas liprin-α1 knockdown itself prevents invasion of MDA-MB-231 breast cancer cells [53]. In addition, liprin-α1 interacts with GIT1 to regulate cell spreading and migration [98]. In metastatic HNSCC and breast cancer cells, liprin-α1 is localized close to the cell edge near vinculin–positive focal adhesions [51, 53]. Liprin-β1 localizes at or close to paxillin-positive focal adhesions, and binding of LAR to liprin-α1 allosterically regulates the liprin-α/liprin-β interaction [80, 99].

Liprin-α1 and -α3 regulate the formation of actin stress fibers by negatively regulating mDia1, a member of the formin protein family and a Rho effector, by displacing the protein from the plasma membrane [100]. Binding of liprin-α3 to mDia1 counteracts RhoA activation and results in reduction of the cellular actin filament formation which leads to decrease in actin filaments in cultured cells [101]. In endothelial cells, liprin-α1 is required for integrin and fibronectin recycling [102]. Liprin-α1 interacts with the cytosolic domain of the α5β1 integrins to promote fibronectin secretion and the recycling of active α5β1 integrins from the *trans-*Golgi network to the surface of endothelial cells, thus allowing Rab11-regulated fibronectin turnover [102].

In zebrafish, morpholino-mediated liprin-α1 knockdown causes the reduction in perivascular fibronectin deposition, and leads to severe heart morphogenesis and cardiovascular defects [102]. This finding is supported by another study in *Xenopus* tadpoles showing liprin-β1 to have a role in lymphatic vascular development and vessel integrity; silencing of liprin-β1 results in edema and dispersed endothelial cells when compared to the controls [103]. The available data demonstrate that liprins are important scaffold proteins regulating adhesion, integrin recycling and protrusive activity of different cell types.

### Liprins and invadosomes

**Fig. 3:**
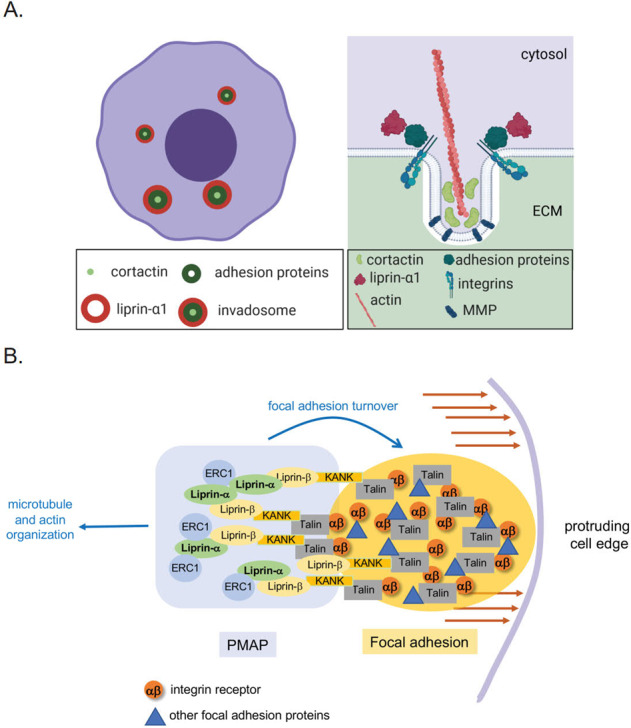
Contribution of liprins to cancer cell motility and invasive capabilities. **A** Simplified model of liprin-α1 contribution to invadosomes and ECM degradation in a context– and cell line–dependent manner. In specific type of cancer cells (on the left) with invadosomes capable of degrading ECM, liprin-α1 is located at the adhesion ring of the invadosome. Liprin-α1 is not necessarily required for ECM degradation per se in these cells, but rather regulates the stabilization and motility of invadosomes. On the right side of the illustration, cross section shows that liprin-α1 is recruited to the invadosome-associated compartments. Cortactin, actin, focal adhesion proteins (such as paxillin, vinculin, and talin) and integrins are recruited to the invadosome, where MMPs play a crucial role in ECM degradation. **B** Model for the interplay between PMAPs and focal adhesions. PMAPs are observed near dynamic focal adhesions at the front of migrating tumor cells, where PMAP proteins are required to promote focal adhesion turnover. A possible link between focal adhesions and PMAPs are represented by the KANK proteins that can interact both with the focal adhesion protein talin, and PMAP proteins liprin beta. PMAPs also link adhesions to the cytoskeleton via PMAP components interacting either with actin or with microtubules.

The term invadosome refers to actin-rich protrusions with degradative properties such as the podosomes commonly found in monocytic cells and the invadopodia present in different types of cancer cells [104–107]. Invadosomes are essential structures in cancer cell invasion, since they are required to degrade the ECM (proteases, MMPs) to promote invasion [105, 108]. Although invadosomes have been mostly studied in vitro, there is evidence supporting the existence of invadosome-like structures in vivo [109, 110]. Invadosomes in non-invasive cancer cells contain podosome-like structures opposite to the actin-based invadosome structures observed in invasive cancer cells, which have gone through EMT. Invadosomes with podosome-like structures are likely precursors of invadopodia [111, 112]. Podosomes function in adhesion as well as in degradation of ECM, and they are formed by a ring of adhesion proteins and an actin-rich core [112–114]. The adhesion ring of invadosomes with podosome-like structures includes the adhesion proteins β1 integrins, paxillin, talin and vinculin, whereas the core includes cortactin and actin [51, 105, 115]. Cortactin is encoded by the *CTTN* gene, which is located in the vicinity of *PPFIA1* at the 11q13 amplicon [116]. It has been suggested that podosomes guide migration by locally anchoring protrusions [114]. Invadopodia on the other hand are irregular, less defined in shape compared to podosomes, with a cortactin– and actin–positive core, but with no adhesion ring [117]. In head and neck squamous cell carcinoma, the actin-based invadosomes differ in their protein content and turnover rate between invasive and non-invasive cells [111]. In metastatic breast cancer cells, liprin-α1 has an important role in stabilizing invadosome function, turnover and degradation of ECM, which are important functions for motile cells [53]. Invadosomes with podosome-like structures are found in poorly invasive HNSCC cells derived from primary tumor [111]. In non-invasive HNSCC cells, liprin-α1 localizes at the outer edge of the adhesion ring or behind the cell edge close to focal adhesions [51] (Fig. 3). Although liprin-α1 is recruited to the adhesion ring of the invadosomes and these structures are able to degrade the ECM, liprin-α1 is not essential for their formation nor degradation [51]. Therefore, the role of liprin-α1 may be rather in the stabilization of these structures in non-invasive cells [51]. In metastatic HNSCC and invasive breast cancer cell lines, liprin-α1 mostly localizes near the leading edge and close to focal adhesions (Fig. 3B). In these cells, liprin-α1 is required for efficient degradation of the ECM, migration, and invasive growth [51–53]. On the other hand, liprin-β1 does not affect ECM degradation in breast cancer cells as shown by knockdown studies [72].While liprin-α1 as well as liprin-β1 have oncogenic pro-invasive properties, liprin-β2 decreases cell migration and the capability of cancer cells to degrade ECM [53, 72].

In Src-transformed NIH-3T3 cells, liprin-α1 is part of a membrane-less invadosome-associated compartment (IAC), near to, but distinct from both the actin-rich core of the invadosomes and the adhesion ring [118]. In these cells, liprin-α1 is not required for the formation of invadosomes, but contributes to their motility and organization: therefore liprin-α1 influences the degradation of the ECM without affecting the recruitment of the transmembrane matrix metalloprotease MT1-MMP to the structures [118]. In vivo studies in xenograft mice have revealed that liprin-α1 promotes the formation of the metastasic lesions in lungs [72]. In addition, upregulation of *PPFIA1* expression indicate higher risk of metastasis relapse in breast cancer patients, specifically in estrogen positive and nodal negative group [119]. The versatile contribution of liprin-α1 on invasion is likely to be explained by the context-dependency of liprin-α1 expression, as invasive and adhesive structures differ in distinct types of cancer cells as explained above. In addition, genetic background, amplification and mutation of the *PPFIA1* gene are probable contributors to the capability of liprin-α1 to modulate cellular events. It is currently obvious that the role of liprin-α1 in cell invasion and invasive growth is cell line- and context-dependent, which would explain previously reported contradicting results on the role of liprin-α1 in cell invasion as discussed below [51–53, 120].

### Liprins in the regulation of invasion and oncogenic signaling

As discussed above, liprin-α1 is important in cell edge dynamics, cell motility, and invasion although its role in invasion has been somewhat controversial [4, 5, 51, 53, 120]. It is now known that in specific non-invasive HNSCC cells, liprin-α1 is required for expansive growth behavior of the cells, while in motile and invasive cells knockdown of liprin-α1 reduces cell invasion and invasive growth [51, 53]. Liprin-α1 knockdown leads to increased expression of vimentin in HNSCC cells, a component of intermediate filaments, as well as disturbances in organization of vimentin filament network in motile or metastatic cancer cells [51]. In support of this data, *PPFIA1* has been reported to be a novel Snail1 target, which suggests that positive regulation of *PPFIA1* expression by Snail1 may contribute to the invasive phenotype of breast cancer cells [121]. It is thus possible that transcriptional regulation of *PPFIA1* by Snail1 may promote vimentin intermediate filament assembly, which is a hallmark of the epithelial-mesenchymal transition [121]. This data underline the importance of liprin-α1 in the regulation and interplay with the cytoskeletal elements relevant to cancer cell motility.

Liprin-α1 suppresses the expression of the transmembrane metastasis suppressor CD82 in HNSCC and breast cancer cell lines [52], and the expression of liprin-α1 and CD82 is negatively correlated in clinical breast cancer samples [122]. CD82 is a direct p53 effector, an important suppressor for metastasis in multiple cancers and an inhibitor of microprotrusions during invasion [123, 124]. CD82 can be modulated by liprin-α1 [52], it is an important player in tetraspanin-enriched membrane microdomains, and it can be activated by post-transcriptional modifications such as glycosylation and palmitoylation [123, 125, 126].

Liprin-α1 interacts with ING4 (inhibitor of growth 4), and positively regulates cell migration in RKO colon carcinoma cells in ING4-dependent manner [127]. Liprin-α1 is expressed at the protrusions, essential for movement in metastatic breast cancer cells [53, 72, 128]. Similarly, liprin-β1 co-localizes at the cell edge with liprin-α1, and contributes positively to tumor cell motility in addition to liprin-α1 [72]. Liprin-β1 interacts with the metastasis-associated protein S100A4, which may modulate liprin-α1/liprin-β1 interaction, thus acting as a component of the LAR-liprin-α1/liprin-β1 network [129].

The N-terminus of liprin-β1 co-immunoprecipitates with the adaptor proteins Kank1 and Kank2, which are suppressors of the proliferation and migration in melanoma cells [130]. Although the functional role of this interaction is not clear, liprin-β1 is likely to contribute to melanoma tumor development. While several studies suggest liprin-β1 to promote tumor progression, liprin-β2 has opposing role in the cancer invasive phenotype, and silencing of liprin‐β2 has no effects on lamellipodia density and stability in MDA-MB-231 cells [72]. Interestingly, ERK2 knockdown leads to increased expression levels of liprin-β2 in MDA-MB-231 breast cancer cells and liprin-β2 knockdown restores the invasive phenotype of ERK2-depleted cells in three-dimensional ECM [75].

Liprins are involved in a number of biological processes important in oncogenic signaling, including cell–cell or cell-substrate junctions, as well as the composition of the cell membrane [5, 51–53, 90, 92, 131]. For example, liprin-α1 expression associates with several pathways important in cancer cell signaling including membrane microdomains and anchoring junctions [52]. Membrane microdomains are cholesterol-containing lipid rafts, which facilitate cellular signaling. These microdomains assemble and disassemble in response to environmental factors during cellular movement, invasion and ECM remodeling [109, 132, 133].

Recently, liprins have been shown to be associated to several proteins that are involved in cancer progression and motility. Pull-down experiments in human bladder cancer cell line T24 showed that liprin-α1 binds to EphA2 and promotes progranulin-dependent motility. Liprin-α1 is abundantly expressed in several bladder cancer cell lines and it strongly co-localizes with EphA2 upon progranulin stimulation [134]. Recent findings in K562 leukemia cells have shown that *PPFIA1* is a direct target of miRNA-181a, whose downregulation is associated with poor response in leukemia. In this work, liprin-α1 overexpression promoted invasive capabilities of K562 cells and transfection of *PPFIA1* siRNA on CML xenograft murine model led to reduced growth of the tumor [135]. Liprin-α4 is upregulated in renal cell carcinoma cell lines, and it is directly activated by binding of the hypoxia-inducible factor 1α (HIF-1α). Liprin-α4 controls the levels of E-cadherin and β-catenin at the epithelial cell junctions [131]. All these data provide evidence of important roles of liprin proteins in oncogenic cell signaling.

## Perspectives

The role of liprins in synaptic signaling in neuronal cells has been extensively studied now for a couple of decades by different in vitro and in vivo studies [1, 6–8, 14, 29, 35, 44] (Table 2). In contrast, the function of liprins in tumor progression has been highlighted in different tumor models only during the last decade [51–53, 72, 127]. While the in vivo data are still fragmentary, in the future the generation of knockout mouse models will provide deeper understanding on the role of these proteins in disease and physiology. Liprins are multifunctional scaffold proteins orchestrating the disassembly and assembly of cellular structures, such as pre- and postsynaptic sites, focal adhesions, and invadosomes [7, 15, 29, 53, 90, 118]. The increasing evidence of liprins’ contribution to tumor cell motility, signaling, recycling of membrane, and ECM proteins such as integrins and fibronectin, and localization of liprins into defined plasma membrane structures reflect their versatile functions in several physiological and pathological processes. Genetic alterations of liprins, in particular the high-level amplifications of *PPFIA1* in HNSCC and breast cancer, point to the critical oncogenic role of liprins in tumor progression. The future research should focus on understanding the role of liprins in those preclinical models which take into account the tumor microenvironment and signaling between tumor cells and stroma. This would enable understanding of the cellular organization and liprin-associated protein complexes and their role in oncogenic signaling. As the interplay between LAR and liprin-α proteins in tumor development and signaling is only partially explored, it is important to find out whether liprin-α proteins contribute to the dephosphorylation activity of PTPases and thus could serve as an interesting drug target or molecular marker. The participation of liprins in LLPS and localization to plasma membrane-associated platforms may reveal in the future interesting insights of liprins in cancer cell signaling. Because the information currently is still limited, further mechanistic studies in the future are required to understand the detailed roles of liprins in oncogenic signaling, tumor progression, and drug related therapy in different tumor types.
